# Genome-wide DNA methylation profiling by modified reduced representation bisulfite sequencing in *Brassica rapa* suggests that epigenetic modifications play a key role in polyploid genome evolution

**DOI:** 10.3389/fpls.2015.00836

**Published:** 2015-10-09

**Authors:** Xun Chen, Xianhong Ge, Jing Wang, Chen Tan, Graham J. King, Kede Liu

**Affiliations:** ^1^National Key Laboratory of Crop Genetic Improvement, National Center of Oil Crop Improvement (Wuhan), College of Plant Science and Technology, Huazhong Agricultural UniversityWuhan, China; ^2^Southern Cross Plant Science, Southern Cross UniversityLismore, NSW, Australia

**Keywords:** *Brassica rapa*, DNA methylation, genome evolution, modified RRBS, polyploid

## Abstract

*Brassica rapa* includes some of the most important vegetables worldwide as well as oilseed crops. The complete annotated genome sequence confirmed its paleohexaploid origins and provides opportunities for exploring the detailed process of polyploid genome evolution. We generated a genome-wide DNA methylation profile for *B. rapa* using a modified reduced representation bisulfite sequencing (RRBS) method. This sampling represented 2.24% of all CG loci (2.5 × 10^5^), 2.16% CHG (2.7 × 10^5^), and 1.68% CHH loci (1.05 × 10^5^) (where H = A, T, or C). Our sampling of DNA methylation in *B. rapa* indicated that 52.4% of CG sites were present as ^5m^CG, with 31.8% of CHG and 8.3% of CHH. It was found that genic regions of single copy genes had significantly higher methylation compared to those of two or three copy genes. Differences in degree of genic DNA methylation were observed in a hierarchical relationship corresponding to the relative age of the three ancestral subgenomes, primarily accounted by single-copy genes. RNA-seq analysis revealed that overall the level of transcription was negatively correlated with mean gene methylation content and depended on copy number or was associated with the different subgenomes. These results provide new insights into the role epigenetic variation plays in polyploid genome evolution, and suggest an alternative mechanism for duplicate gene loss.

## Introduction

Polyploidy, where more than two complete sets of chromosomes reside within the same nucleus, is both pervasive and ancient in most eukaryotic lineages and also is particularly prevalent in plants (Jiao et al., 2011; Proost et al., 2011). Polyploidization results in gene duplication, redundancy, and increased genome size, following which a dynamic polyploid genome will experience extensive and rapid genome restructuring, genome downsizing and ultimately genetic “diploidization” at many loci (Soltis and Soltis, 1999; Wolfe, 2001). The mechanism of diploidization remains a mystery, although the loss of duplicated copies from the genomes of ancient polyploid species known as “fractionation” has been considered a major force for plant genome evolution (Langham et al., 2004). Interestingly, gene losses within different genomes are usually unequal, with one of the duplicated genomes consistently losing significantly more genes than the others. This bias in gene loss was first observed in *Arabidopsis* (Thomas et al., 2006) and more recently in maize (Woodhouse et al., 2010; Schnable et al., 2011) and *Brassica rapa* (Wang et al., 2011; Tang et al., 2012), and is probably a general characteristic of major eukaryote lineages where paleopolyploidy has been involved (Sankoff et al., 2010).

In maize, a monocotyledon species that experienced tetraploidy 5–12 million years ago, biased gene losses between two complete subgenomes are both ancient and ongoing among diverse inbreds, primarily resulting from a mechanism of short deletions (Woodhouse et al., 2010). In particular, genes from the genome that has experienced less gene loss are expressed at a higher level than those from the other subgenome. This suggests that the bias in gene loss between the subgenomes may be the result of selection against loss of the genes responsible for the majority of the expression within a duplicated gene pair (Schnable and Freeling, 2011; Schnable et al., 2011). A corollary of this is that genes with lower expression levels are more readily deleted, since their removal is less likely to lower fitness, and so they escape purifying selection. Differential epigenetic marking of the genomes within an allopolyploid has been suggested as a mechanism underlying differential expression of genes retained in different subgenomes (Schnable et al., 2011; Diez et al., 2014).

Cytosine methylation is a major epigenetic mark and plays an important role in chromatin conformation, in silencing different types of repetitive sequence and in regulating transcription (Bird, 2002). In plants, methylated cytosine residues are observed at cytosine bases in all sequence contexts, including symmetric CG and CHG (where H = A, T, or C) and asymmetric CHH (Henderson and Jacobsen, 2007). Methylated cytosine (^5m^C) is especially pervasive in intergenic regions but also within protein-coding regions, where it is typically limited to the CG context (Cokus et al., 2008; Lister et al., 2008). *De novo* methylation in plants is catalyzed by DOMAINS REARRANGED METHYLTRANSFERASE2 (DRM2) but maintained by different pathways, with ^5m^CG maintained by DNA METHYLTRANSFERASE1 (MET1), ^5m^ CHG by CHROMOMETHYLASE3 (CMT3), and asymmetric ^5m^ CHH dependent on persistent *de novo* methylation by DRM2 (Chan et al., 2005; Law and Jacobsen, 2010). Small RNA (sRNA)–mediated DNA methylation (RdDM) can target methylation of transposable elements (TEs) in many eukaryotic lineages and contribute to limiting TE proliferation (Almeida and Allshire, 2005). However, silencing of TEs may have collateral effects on the transcription of nearby genes, which can lead to preferential loss of methylated TEs from gene-rich chromosomal regions (Hollister and Gaut, 2009).

Cultivated *Brassica* species belong to the monophyletic *Brassiceae* tribe within the dicotyledon family *Brassicaceae*. Diploid *Brassica* genomes were hypothesized to have been triplicated and confirmed by many molecular marker and cytologenetic evidence (for review, Prakash et al., 2009) as well as recent whole genome sequencing of *B. rapa* (Wang et al., 2011) and *B. oleracea* (Liu et al., 2014; Parkin et al., 2014). Whole genome sequencing provides an opportunity to identify different subgenomes within the *B. rapa* genome by synthetic comparison between *B. rapa* and *A. thaliana*. Three subgenomes have been proposed, each with significant deviations from equivalent gene frequencies. The least fractionated (LF) subgenome retains 70% of the genes found in *A. thaliana*, the medium fractionated (MF1) 46%, and the most fractionated (MF2) 36% (Wang et al., 2011). Based on examining short exonic deletions in retained *Brassica* genes, Tang et al. (2012) further revealed that subgenome II (MF1) had more recent deletions than subgenome I (LF) or subgenome III (MF2), which suggested that a two-step process of genome fractionation had indeed occurred.

Here, we want to ask if biased gene loss during *B. rapa* genome evolution has been driven by, or has consequences for, epigenetic processes. In other words, if there are significantly difference in DNA methylation for genes of different copy numbers or with different subgenomes. We firstly generated a genome-scale DNA methylation profile for *B. rapa* using modified RRBS, and then compared this profile with gene transcription data from RNA-seq. We found that genes in the different subgenomes display a hierarchical level of cytosine methylation and transcription. In particular, the singleton genes have a significantly higher level of DNA methylation than genes with two or three paralogues, and are expressed at a lower level.

## Materials and methods

### Samples and DNA extraction

A semi-winter type *B. rapa* var. oleifera (2*n* = 20, AA genome, genotype 3H120) was used in this study. This inbred line had previously been used as one of the parents for new allopolyploid synthesis in order to investigate genetic and epigenetic changes following hybridization and genome doubling between different *Brassica* diploid species (Cui et al., 2012). Because all hybrid immature embryos were cultured and new plants were developed and conserved on MS medium, the parent plants used for hybridization were then also conserved by tissue culture. Briefly, the plant was firstly sub-cultured on MS medium with 1.5 mg/liter 6-benzyl aminopurine (6-BA) and 0.25 mg/liter a-naphthalenacetic acid (NAA) to generate sufficient cloned plantlets, which were then successively cultured on MS agar medium. Young leaves were collected from the young plants on MS medium and immediately frozen in liquid nitrogen. Genomic DNA was extracted from ~100 mg tissue using the DNeasy Plant Mini Kit (Qiagen, Valencia, CA), and DNA content was quantified by Qubit HS dsDNA kit.

### Bisulfite treatment and sequencing library construction

Approximately 500 ng gDNA was simultaneously double digested using *SacI* (GAGCTC) and *MseI* (TTAA) (Fermentas) in a reaction volume of 25 μl. The reaction mixture was first incubated at 37°C for 6 h, and then at 65°C for 90 min. Sac_meAD and Mse_meAD adaptors (Figure S1) were annealed using the program: 94°C gradually decreased to 65°C with −0.5°C every 10 s, then kept at 65°C for 10 min, 56°C for 10 min, 37°C for 10 min, and 22°C for 10 min. Restriction fragments were ligated to the Sac_meAD and Mse_meAD adaptors with unique index sequences. The ligation reaction was carried out in 50 μl at 16°C overnight with 25 pmol Sac_meAD and Mse_meAD adaptors, and 50,000 Units of T4 DNA ligase (NEB). The resulting ligates with different index sequences were mixed and concentrated using a PCR purification kit (Qiagen, Valencia, CA) and fragments between 250 and 500 bp were cut from a 2% agarose gel and purified with the Qiagen gel purification kit (Qiagen, Valencia, CA). ~500 ng recovered products were subjected to two successive treatments with sodium bisulfite using EpiTect Bisulfite kit (Qiagen, Valencia, CA) following the manufacturer's instructions. After a final purification using the PCR purification kit, 5 μl bisulfite-converted ligates were amplified by 18 PCR cycles with the following reaction composition: 1 × Taq buffer, 3.5 mM MgCl_2_, 0.4 mM dNTPs, 1 U Taq DNA polymerase (Fermentas), and 5 pmol Illumina PCR primers (Chen et al., 2013). The enriched library was purified with Qiagen gel purification kit, and quantified by Qubit HS dsDNA kit. The library was sequenced on Hiseq 2000 platform according to the manufacturer's instructions.

### Sequence filtering and alignment

After parsing reads into different subsets based on the index sequences, the first 75 bp of paired-end (PE) reads were retained, and the residual enzyme recognition sequences trimmed. Low-quality PE reads containing more than 5% of nucleotides with Phred quality value < 30 were filtered by the IlluQC.pl script included in NGSQCToolkit_v2.3 program suit (Patel and Jain, 2012). The remaining high-quality reads were mapped against the *B. rapa* var. *pekinensis* Chiifu-401-1 reference genome sequence (v1.2) (Wang et al., 2011) using Bismark_v0.7.4 software (Krueger and Andrews, 2011) in a non-directional manner with a maximum of 1 bp mismatch in multi-seed alignment. Only uniquely mapped reads were retained for further analyses.

### Calling methylated loci

Overlapping sequences of paired-end reads were ignored to prevent mis-calculating the level of methylation. In order to call a methylation score for each potential CG, CHG, and CHH site, high quality cytosines (≥ 20 phred quality score) within methylation loci having at least 10-fold coverage were extracted by the methCall.pl script (https://code.google.com/p/methylkit/source/browse/exec/methCall.pl?r=dd63fb95d718356e94c46ef2885d4110b385297d). Gene and TE annotations were obtained from BRAD (http://brassicadb.org/brad/). Tandem and inverted repeats were detected using Tandem Repeat Finder and Inverted Repeat Finder software packages following default parameters (Benson, 1999; Warburton et al., 2004).

### SNP detection using a modified ddRAD protocol

To remove the influence of nucleotide variations during the calling of methylated loci, modified ddRAD sequencing was performed simultaneously according to the protocol published previously (Chen et al., 2013). After sequence trimming, 75 bp paired-end clean data were aligned to the *B. rapa* reference genome sequence (v1.2) using Bowtie2 software with a maximum of one mismatch (Wang et al., 2011). SNP calling was performed by Samtools software with the parameters of at least one coverage with phred quality of ≥ 20 (Li et al., 2009; Langmead and Salzberg, 2012). Finally methylation sites disrupted by SNPs in the 3H120 genome were excluded from further analyses.

### Methylation level distributions analysis

To examine the genome scale distribution of methylated and repeat sequences, we plotted the average methylation level and length of repeat sequences across each chromosome using a 200 kb sliding windows with 100 kb overlap. The length of genes and transposons were variable, hence we plotted the methylation level using a sliding windows corresponding to 10% of the length of specific genes or transposons. Promoter regions were defined as the 200 bp immediately upstream of the transcriptional start site (TSS) of each gene, and upstream and downstream of genes and transposons were defined as 1 kb 5′ and 3′.

### Gene expression analysis

RNA-seq data from different organ and tissues of 3H120 (Zhang et al., unpublished data) and Chiifu-401 (Tong et al., 2013) were used for gene expression analysis. Genes were firstly classified into different subgenomes or groups with different copies according to published *B. rapa* reference genome (Wang et al., 2011). Then, in each group, genes were assigned into three classes of high (RPKM/FPKM > 50), medium (5 < RPKM/FPKM ≤ 50) and low (RPKM/FPKM ≤ 5) transcription (Tong et al., 2013) (Table S1).

## Results

### Representative DNA methylation profile for the *B. rapa* genome

In order to generate a representative profile of the global DNA methylation in *B. rapa*, a modified RRBS protocol was used (Figure S1), yielding a total of 2.3 Gb PE100 (100 bp paired-end) sequence data. Following trimming and filtering, 1.28 Gb (corresponding to 8.56 million PE75 reads) were retained for subsequent analyses, of which 0.55 Gb (42.9%) could be successfully and uniquely aligned to the *B. rapa* reference genome using Bismark (Krueger and Andrews, 2011). These data were used to call a methylation level for each CG, CHG, and CHH site. Because SNPs between the reference *B. rapa* Chiifu-401 and the 3H120 genotype could potentially interrupt the methylation calling, we performed standard non-bisulfite sequencing of the 3H120 genome following the modified double digest Restriction-Site Associated DNA sequencing (ddRADseq) protocol (Chen et al., 2013). After sequence trimming and filtering, a total of 3.2 million PE75 (0.48 G) high-quality reads were collected. These sequences were aligned to the reference *B. rapa* Chiifu-401 genome by Bowtie2, and a total of 36,836 candidate SNPs were detected using Samtools. Of the methylated loci that had been called, 1031 CG, 736 CHG, and 1886 CHH sites were disrupted by these candidate SNPs and so were excluded. Finally, a total of 0.26 million CG, 0.27 million CHG, and 1.05 million CHH loci were recovered following alignment, which respectively accounted for 2.24, 2.16, and 1.68% of the total loci in the *B. rapa* genome. Taking those with a minimum sequencing depth of 10, the frequency of the three 5^m^C contexts detected represented 0.64% CG, 0.59% CHG, and 0.43% of all CHH loci and were used for subsequent analyses. In order to determine whether the relative proportion of CG, CHG, and CHH sites enriched in genic and transposon regions was consistent with those in other genomes (Figure S2A), we performed an *in silico* mRRBS (simulation restriction enzyme digestion analysis) of the *B. rapa var. pekinesis* (Chiifu-401-1) and rice (*Oriza sativa L.SSP indica* 93-11) genome and calculated the relative proportion of each of the three methylation contexts (Figure S2B). We found a similar proportional representation of the frequencies at the whole genome level. This gave us confidence that the modified RRBS protocol we adopted would provide a reliable representation of DNA methylation across the *B. rapa* genome.

### DNA methylation landscape of *B. rapa* genome

Our sampling of DNA methylation in *B. rapa* indicated that 52.4% of CG sites were present as ^5m^CG, with 31.8% of CHG and 8.3% of CHH (Table S2). At single base resolution, 92.5% of CG sites were either unmethylated or highly methylated (90–100%), whereas 71% CHH sites were either unmethylated or hypomethylated (0–10% as ^5m^CHH per site). For the CHG context, 51.8% of sites were hypomethylated and 10.3% sites were highly methylated, with a more uniform distribution between 10 and 90% (Figure S3). These differences may result from the distinct genetic control under which context methylation arises and is maintained (Law and Jacobsen, 2010; He et al., 2011).

**Figure 1:**
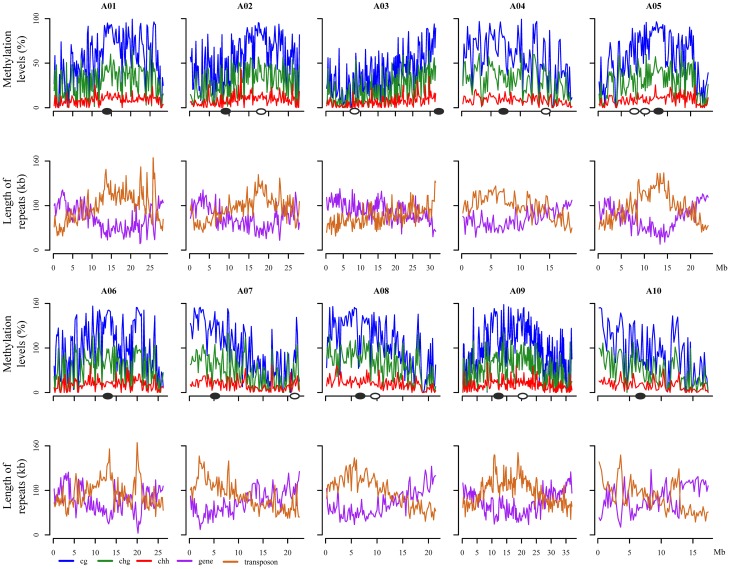
**Genome-wide distribution of methylation level, transposon and gene contents along 10 ***B. rapa*** chromosomes**. The X axis indicates the physical distances, and Y axis indicates the average methylation level or length of repeat in each 200-kb windows. Solid oval circles stand for the position of real centromeres, blank oval circles stand for the position of ancient centromeres (Wang et al., 2011; Cheng et al., 2013). CG, CHG, and CHH (where H = A, T, or C) indicates methylated cytosine residues in different sequence contexts.

The distribution of the mean methylation level for each of the three contexts (CG, CHG, and CHH) in *B. rapa* was calculated for each chromosome, with a sliding windows of 200 kb (Figure 1). CG methylation was consistently higher than CHG and CHH, with CHH methylation lowest throughout the genome. We found that the average CG, CHG, and CHH methylation distributions were highly correlated, despite being maintained through distinct genetic mechanisms. In order to study the relationship between the level of DNA methylation and repeat elements, transposons, tandem and inverted repeat sequences, and the frequency of these sequence classes was plotted in sliding 200-kb windows across each chromosome (Figure 1). This indicated that higher levels of DNA methylation level were associated with regions enriched for repetitive sequences, and low levels of methylation distribution were dispersed in regions enriched for genes. We found a more complex distribution of methylation across chromosomes than that observed in *Arabidopsis* (Cokus et al., 2008), although there remained a positive correlation with repeat elements and a negative correlation with gene density (Figure 1). These results indicated that overall the distribution of DNA methylation largely reflects the relative density of transposons, retrotransposons, and other repetitive sequences.

In *Arabidopsis*, hyper DNA methylation has been associated with pericentromeric regions, primarily as a result of enrichment of diverse repetitive sequences (Cokus et al., 2008). Within the *B. rapa* genome, 18 of 21 paleocentromeric regions had been detected, including 10 extant *B. rapa* centromeres (Wang et al., 2011; Cheng et al., 2013). As expected, we found extensive DNA methylation in these repeat-rich pericentromeric regions for most chromosomes. However, a lower level of DNA methylation was associated with the pericentromeric region of chromosome A02. This reflects the reduced density of repeat sequences mapped in the vicinity of the A02 centromere. Interestingly, high levels of DNA methylation were found distributed around the eight remaining ancestral centromere regions, particularly in chromosome A02 (Figure 1).

### Patterns of DNA methylation in different components

**Figure 2:**
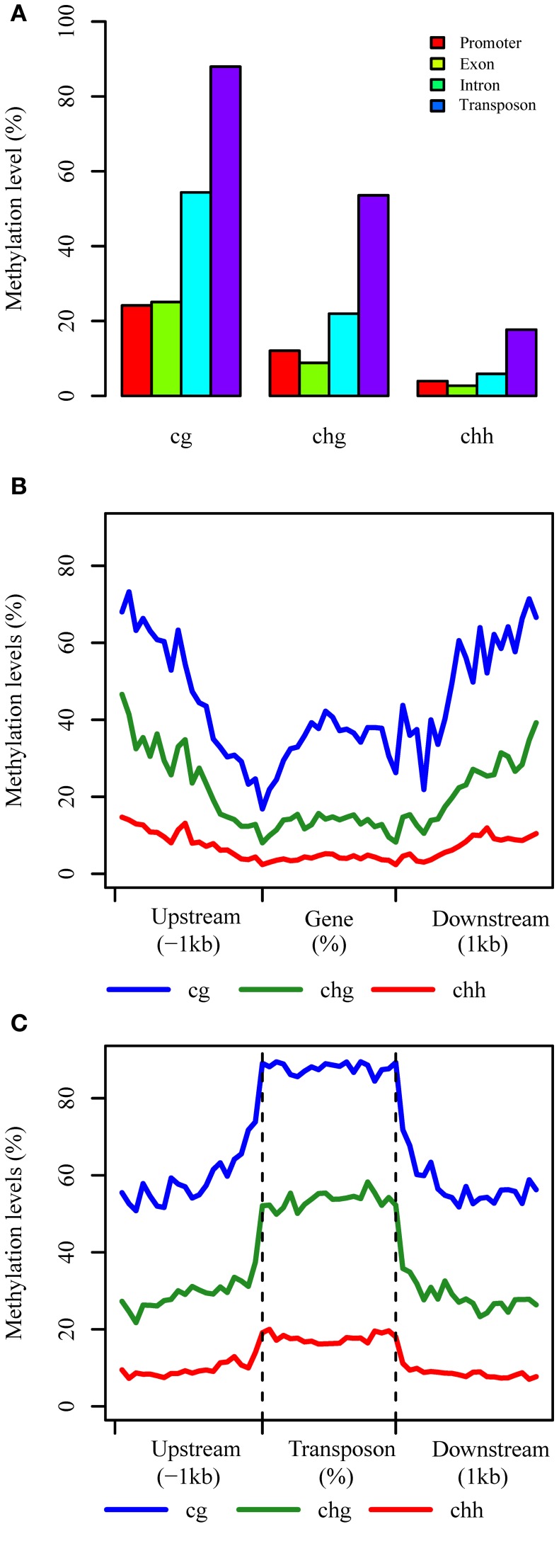
**The distributions of each context in different components**. **(A)** Average methylation level in different components, and promoter regions are defined as 200 bp region before TSS; **(B,C)** Methylation patterns of genic and TE regions, respectively at base-pair resolution.

We characterized the methylation patterns of transcribed genes and TEs in *B. rapa*, by comparing the average DNA methylation level for each context (Figure 2A). In genic regions, a greater than two-fold methylation was detected in introns (CG 54.4%, CHG 22.0%, and CHH 5.9%) compared to exonic regions (CG 25.1%, CHG 8.8%, and CHH 2.7%). The level of DNA methylation in exon sequences differed from intron sequences for each context, while they were similar in promoter regions (defined as 200 bp upstream of transcriptional start site, TSS) (Figure 2A). An approximately two-fold higher methylation was detected in TE regions (CG 88.0%, CHG 54.0%, and CHH 17.7%) compared with introns, which suggests more extensive methylation in transposons compared to genic regions.

We then characterized the methylation patterns in genic and TE regions at base-pair resolution for each context. This revealed a similar pattern of CG, CHG, and CHH methylation for these genomic components as found in *Arabidopsis*, although with a higher level of methylation in genic regions (Figure 2B). The levels of CG methylation in genic regions decreased from 1 kb upstream to the TSS and increased throughout the transcribed region before decreasing again up to the transcription termination region (TTR), where after it increased throughout the downstream region. A contrasting pattern was observed for non-CG methylation, with relatively low levels in the gene body compared to upstream and downstream regions, and the lowest levels of methylated detected around the TSS and TTR regions, which in *Arabidopsis* are more highly correlated with gene expression (Cokus et al., 2008), Rice (Li et al., 2012), and Soybean (Song et al., 2013).

An important function of DNA methylation in plant genomes is to modulate the silencing of transposon elements. We found that DNA methylation in TEs is indeed higher than genic regions, and also higher than upstream and downstream regions of TEs. Interestingly, the level of DNA methylation changes dramatically at the TE boundaries, with a sharp increase and decrease around the transcription start and end sites, although DNA methylation levels are maintained relatively consistently across the transposons (Figure 2C).

### Differential methylation of RNA PolII transcribed genes

**Figure 3:**
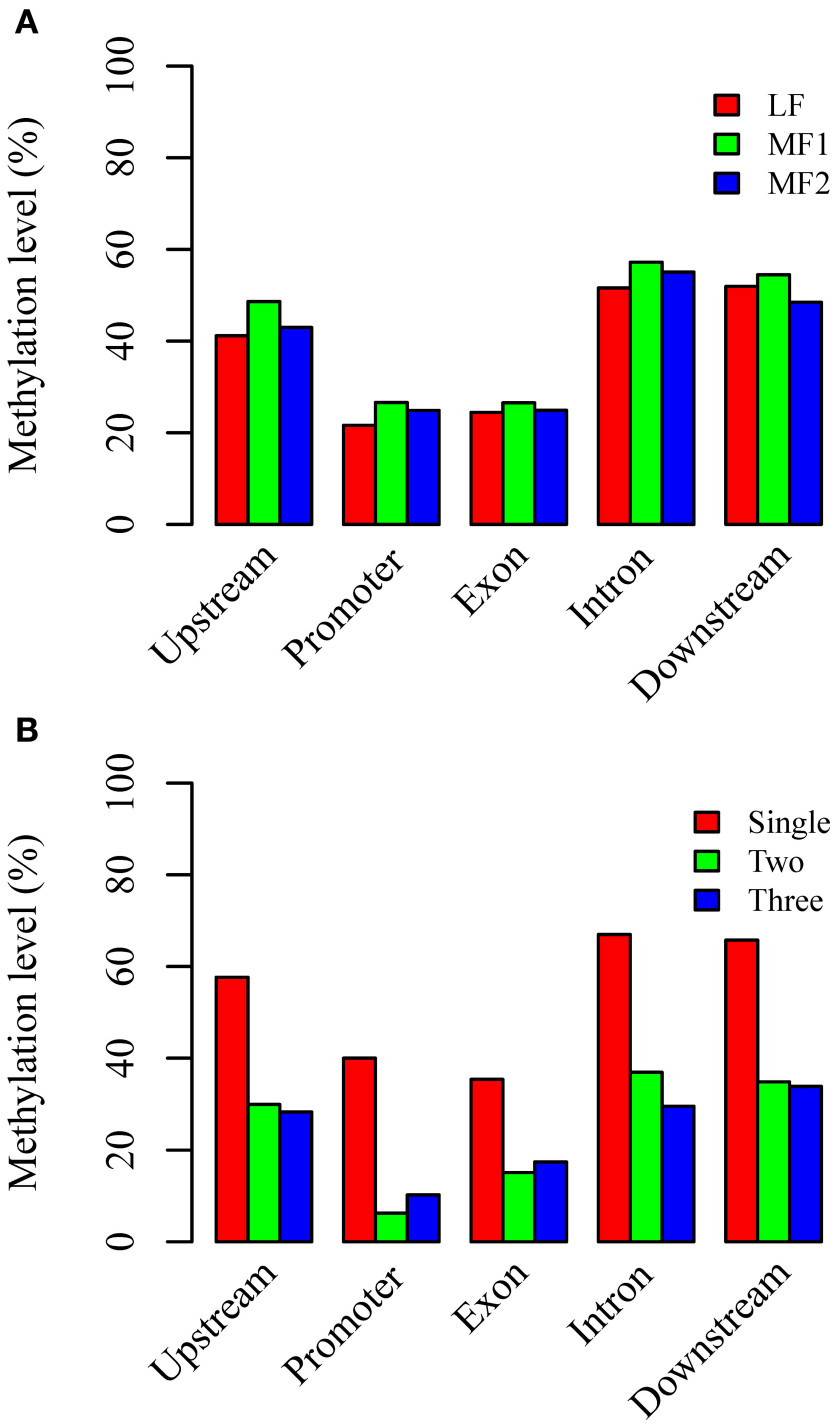
**Average CG methylation levels in different components of genic regions (A) between three subgenomes and (B) between different copy genes**. Whereas, promoter regions are defined as 200 bp regions before TSS site, upstream or downstream regions are defined as 1000 bp before TSS or after TTR sites.

**Figure 4:**
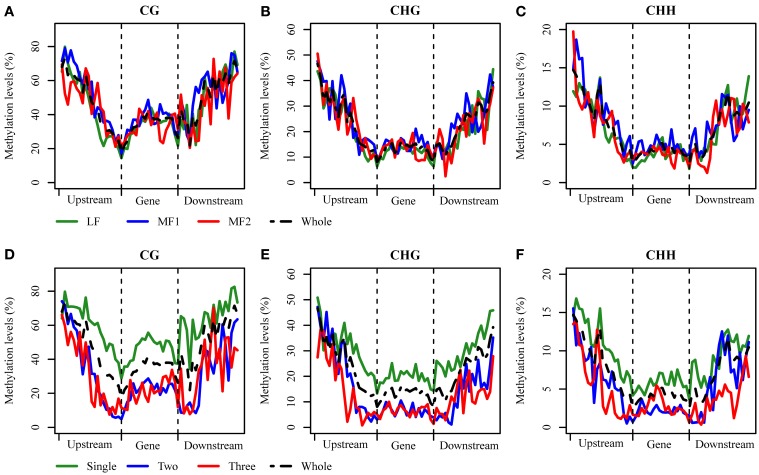
**DNA methylation distributed across genic regions at base-pair resolution**. **(A–C)** between three subgenomes for CG, CHG, and CHH context and **(D–F)** between different copy genes for CG, CHG, and CHH context.

Ancestral duplication events have resulted in the mesopolyploid *B. rapa* having triplicated genomic segments, each of which have undergone different levels of gene loss (Wang et al., 2011). These three subgenomes have therefore also been defined according to their ratios of differential gene loss. The phenomenon is apparent from the retained duplication of some genes and single-copy status of others, although the mechanisms driving this are remain unclear. Three subgenomes, LF, MF1, and MF2 have been identified in the *B. rapa* whole genome sequence (Wang et al., 2011; Cheng et al., 2013). We generated profiles of DNA methylation in genic regions belonging to each subgenome. The average methylation for RNA PolII transcribed genes differed significantly between sub-genomes for each context, with CG: 29.71% (LF), 33.68% (MF1), and 31.76% (MF2); CHG: 11.23, 13.86, and 12.69%; and CHH are 3.46; 4.48, and 3.60% (χ^2^ > 6.63, *P* < 0.01 in each pairwise comparison). We also investigated the average methylation in each sequence context for upstream, promoter, exon, intron, and downstream components of protein coding genes (Figure 3A, Figures S4A, S5A). It was clear that the average methylation level of genic regions in MF1 was higher than LF and MF2, apart from downstream CHH, where LF is highest. Methylation levels in LF and MF2 appeared very similar. For the CG and CHG context, MF2 is slightly higher than LF, apart from the downstream region, although for the CHH context, LF appears slightly higher than MF2 in all components. When we classified the methylated loci according to the three subgenomes and plotted the methylation level across genic regions, we also found that MF1 was clearly more methylated than the other two subgenomes, although there were few systematic differences between LF and MF2 (Figures 4A–C). Overall, we conclude that genes in the MF1 subgenome are most highly methylated and in the LF subgenomes least methylated.

We also characterized average methylation levels in different component regions of single-copy, duplicated, and triplicated genes (Figure 3B; Figures S4B, S5B). For the CG context, we found that methylation in promoter regions of single-copy genes was 6.4-fold higher than duplicated genes and 3.9-fold higher than triplicated genes (Figure 3B). For duplicated and triplicated genes, a similar difference was observed, although it was inconsistent with respect to different sequence components. For example, although there is less methylation in promoter and exon regions of duplicated compared with triplicated genes, we found the converse for other components. In contrast, the pattern of methylation was relatively consistent for the CHG and CHH contexts, with only some differences appearing in promoter regions, whereas for the CHH context there were similar levels of methylation in these regions for both duplicated and triplicated genes (Figures S4, S5). In contrast to the differences in methylation observed between subgenomes, when we classified and plotted the methylated loci according to different-copy genes, very clear differences were detected across the genic regions between single-copy and duplicated genes (Figures 4D–F). We also observed that for the gene body, regions before the TSS and after the TTR were mostly differentially methylated, and are likely to be most responsive with respect to transcriptional repression or activation.

**Figure 5:**
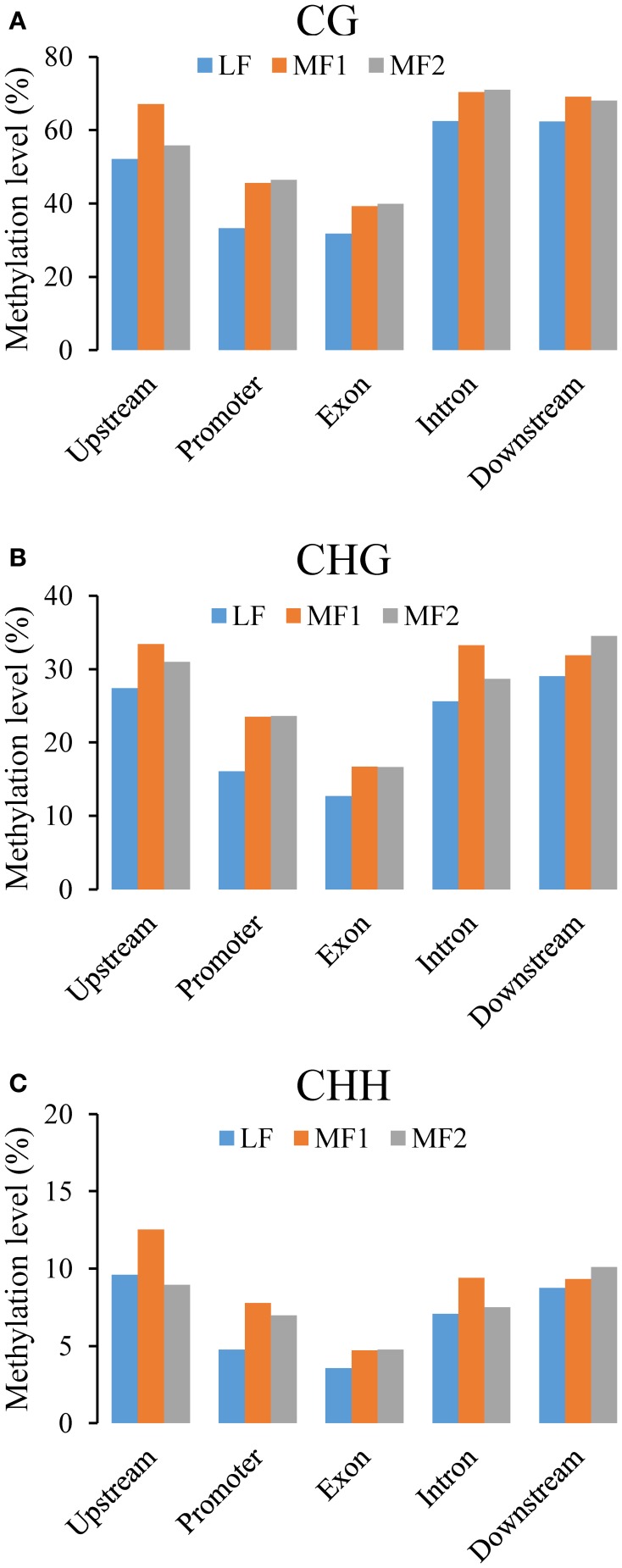
**Average methylation level in single-copy genes between three subgenomes for (A) CG, (B) CHG, and (C) CHH context**. Whereas, promoter regions are defined as 200 bp regions before TSS site, upstream or downstream regions are defined as 1000 bp before TSS or after TTR sites.

We further analyzed the DNA methylation of single-copy and duplicated genes in each subgenome (Figure 5). For single copy genes in MF1 and MF2, the level of methylation is either very similar or MF1 > MF2 apart from downstream regions. However, duplicated genes did not appear to have a systematic difference between the three subgenomes, although MF1 was clearly hypermethylated in promoter region for the CG and CHG contexts (Figures 5A,B). Overall, we observed a consistent difference in level of methylation level in single-copy genes between LF and the other two subgenomes, although this was not apparent in duplicated genes (Figures S6, S7). Thus, we considered that differential level of DNA methylation between different the subgenomes was primarily accounted for by the difference in single-copy genes.

### Differential transcription in relation to DNA methylation, ancestral subgenome, and gene copy number

**Figure 6:**
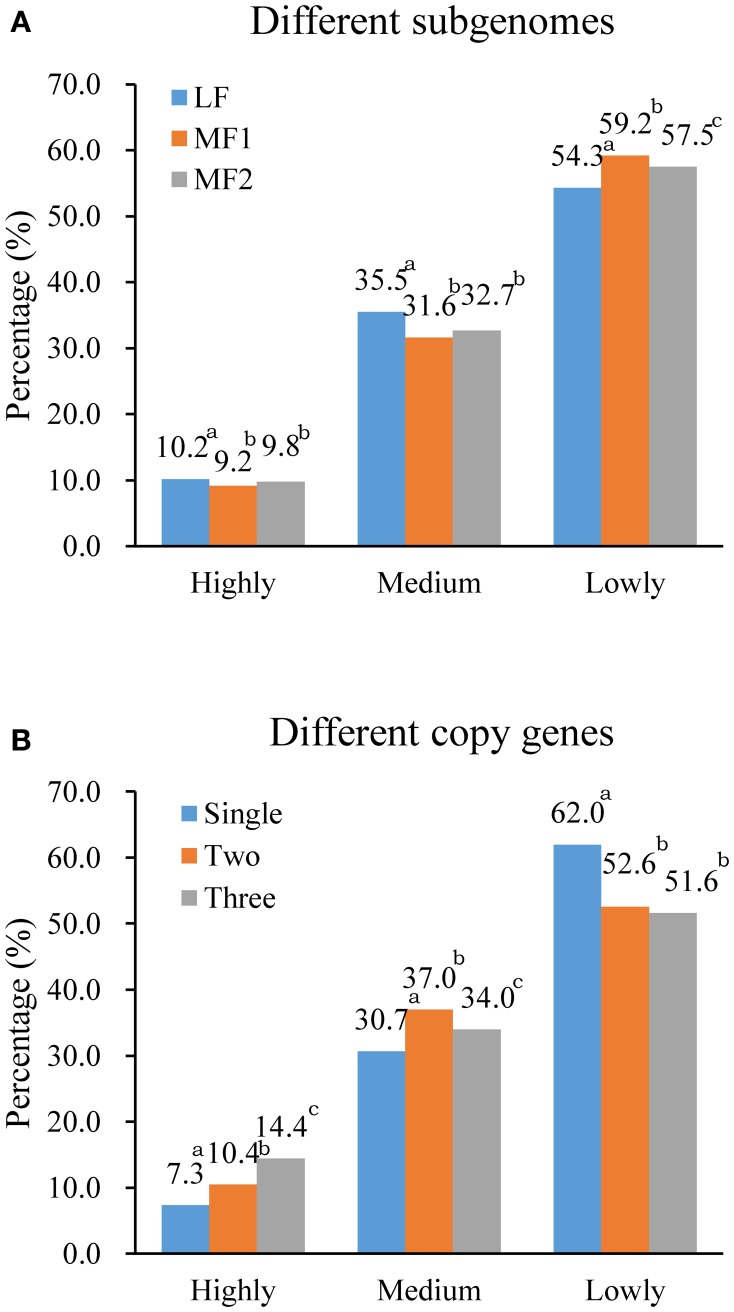
**Differential gene expression (A) between three subgenomes and (B) between different copy genes**. Expressed genes were collected into lowly (≤ 5), medium (5 < RPKM ≤ 50) and highly (> 50) groups according to RPKM value. Superscript a, b, c indicated the significance of gene expression level with different copies or within different subgenomes.

In, 3H120, for leaf tissue (Figure 6A), we found that more genes were included in the high and medium transcription group with fewer genes in the low group in LF than in MF1 and MF2, which indicated that genes in the LF subgenome are significantly expressed at a higher level. In contrast, for MF1 fewer genes were included in the high and medium group and more genes had low levels of transcription than in LF and MF2. Genes in MF2 showed a medium transcription level between MF1 and LF. Thus, the, average transcription level in three subgenome fitted the LF > MF2 > MF1 relationships. We also characterized transcription in leaf tissue for different gene copies (Figure 6B) and found that only 7.3% of single-copy genes were highly expressed, compare to 14.4% three-copy genes and 10.4% two-copy genes. In the medium expression group, the percentage of two-copy genes is highest (37.0%), while lowest (30.7%) for single-copy genes. Compared to two- or three-copy genes, the majority of single-copy genes had a relatively lower level of transcription.

This pattern appeared to be consistent with results for silique tissue from *B. rapa* 3H120 and in different tissues from Chiifu-401-42 (data from Tong et al., 2013) (Table S3). We therefore deduce that the level of transcription for duplicated genes is significantly dominant compared to single-copy genes, and that this is consistent with the significantly higher DNA methylation of single-copy genes compared with duplicated genes. In conclusion, we find strong evidence that the relationship between DNA methylation and level of transcription is more dependent upon the copy number of genes than it is between different subgenomes of *B. rapa*.

## Discussion

Cytosine methylation is an epigenetic modification of DNA that is also associated with histone modification and nucleosome positioning. In higher plants, methylation modification of cytosine (5^m^C) is present in CG, CHG, and asymmetric CHH sequence contexts (where H is A, C, or T) (Henderson and Jacobsen, 2007). Whole-genome bisulfite sequencing (WGBS), including MethylC-seq (Lister et al., 2009) and BS-seq (Cokus et al., 2008; Laurent et al., 2010), is the most comprehensive method for genome wide methylation analysis giving single-cytosine methylation resolution and direct estimates of the proportion of molecules methylated. However, the method requires deep re-sequencing of the entire genome, which is still expensive for complex crop genomes.

The common RRBS protocol tends to enrich CG-rich sequences in the genome due to the usage of restriction enzyme MspI. For mammalian genomes, this enables the majority of CG islands, promoters or other relevant genomic regions in to be captured with limited sequence data (Gu et al., 2011). However, plants have a different pattern of CG distribution, “mosaic” methylation across genome and lack of high unmethylated CG islands (Feng et al., 2010). Moreover, this method is also limited by the uneven distribution of captured regions across chromosomes and inability to represent all sequence components, rendering it unsuitable for profiling genome-wide DNA methylation. In our previous study, SacI/MseI RE combination was used to construct a modified ddRAD library for SNP calling in a doubled haploid (DH) population, and the targeted fragments were found to be evenly and randomly distributed across the *Brassica* genome (Chen et al., 2013). Hence a new double enzyme digested RRBS method was used here to interpret the global DNA methylation at single-base resolution in *B. rapa*. The results show that the percentage of CG, CHG, and CHH loci located in genic regions was consistent between enriched targeted regions using modified RRBS protocol and whole-genome methylation loci. Coupled with an *in silico* double digestion analysis of the rice genome, we were able to confirm the applicability of this modified RRBS approach. Due to the advantages of cost effectiveness and simplicity, modified RRBS is well-suited for DNA methylation profiling of large natural populations or for construction DNA methylation genetic maps (Long et al., 2011).

The methylation ratios observed for each context in *B. rapa* were much higher compared to those reported for the DNA methylome of *Arabidopsis* (Cokus et al., 2008). This may have resulted from the higher level of repeat sequences, especially with for the additional transposable elements in *B. rapa* genome compared with *Arabidopsis* (Wang et al., 2011). In comparison with recently released *B. oleracea* (C genome) and *B. napus* (AC) methylomes, our *B. rapa* analysis has indicated a similar percentage of mCG but higher levels of mCHG and mCHH. It is perhaps surprising that the methylation level of the three contexts are similar or higher in this *B. rapa* compared with *B. oleracea* which contains significantly more repeat sequences, specific recent expansion of the *Bot1* CACTA transposon family (Alix et al., 2008) and a reported higher level of methylation (Liu et al., 2014). In *B. napus*, the C_n_ subgenome was also found to have a higher methylation level than that of the A_n_ subgenome (Chalhoub et al., 2014). Our results suggest a slight increase on this for the *B. rapa* methylome. This may have resulted from the different methods used, with only 2% of the complete set of genome loci recovered in this analysis. We also note that our DNA was isolated from tissue cultured plants. Tissue culture has been found to induce DNA methylation changes in different plant species (Kaeppler and Phillips, 1993; Hang et al., 2009; Linacero et al., 2011; Gonzalez et al., 2013; Stroud et al., 2013; Stelpflug et al., 2014), with the degree and the direction of methylation changes varying with different tissues or cell-types and culture methods. In *Arabidopsis* suspension culture, it was found that a prevalence of DNA methylation increases in genic regions as opposed to losses (Bednarek et al., 2007; Tanurdzic et al., 2008). In recent whole genome level surveys of maize and rice, it was found that losses of DNA methylation following tissue culture are more common than gains of DNA methylation. Meanwhile, it was also found that the bulk of the methylome were not affected, although a subset of genomic regions exhibit altered DNA methylation levels (Stroud et al., 2013; Stelpflug et al., 2014). Although we do not know the true effect on cultured *B. rapa* here, the culture methods used here were similar to those of mazie and rice (Stroud et al., 2013; Stelpflug et al., 2014). Moreover, an earlier methylation sensitive amplification polymorphism (MSAP) survey of the *B. napus* genome (Long et al., 2011) found that a very high proportion of parental methylation alleles were conserved intact in segregating lines maintained through five meiosis following initial tissue culture of the F1 line.

A “two-step theory” for paleohexaploid *B. rapa* formation is sufficient to explain why MF1 and MF2 are more fractionated than the LF subgenome (Wang et al., 2011; Cheng et al., 2013). It has been proposed that MF1 and MF2 are of similar age and first came together to form a tetraploid, with subsequent inclusion of LF to form the ancestral hexaploid. MF1 and MF2 may therefore have resided in the same nucleus for a longer period of time than LF, which is then relatively less fractionated than the first two. We were interested to establish whether the relative methylation level of these different subgenomes may provide some insights into an epigenetic basis for complex genome evolution (Schnable and Freeling, 2011; Diez et al., 2014; Woodhouse et al., 2014). As anticipated, we found that genes in LF had the lowest level of methylation, at least for CG and CHG contexts, corresponding to the highest level of gene transcription. These results are consistent with those in *B. oleracea*, in which lower methylation levels were found in the least fractionated genome (Parkin et al., 2014), although the levels for MF1 and MF2 were reversed with respect to *B. rapa*. We found that for *B. rapa* the levels of methylation were inversely related to gene expression for each subgenome (DNA methylation: MF1 > MF2 > LF; Gene expression: LF > MF2 > MF1), with a bias to fractionation in MF2 compared with MF1 that was not consistent with the pattern of epigenetic marks. It is most likely that MF1 and MF2 came together and contributed to an early tetraploid karyotype (at least 5–9 MYA) (Wang et al., 2011), and over a long period of time, MF2 emerged as the subdominant genome, carrying a higher load of DNA methylation and associated lower level of gene transcription, which resulted in greater gene loss compared with MF1. However, following the incorporation of LF, the original methylation status may have been modified by epigenetic reprogramming in the early stages of the new polyploid formatting (Lukens et al., 2006; Gaeta et al., 2007; Szadkowski et al., 2010; Cui et al., 2013). This provides a realistic explanation for the resulting MF1 > MF2 > LF hierarchy of methylation. Subsequently, a new cycle of gene loss is likely to have arisen, based on the revised pattern gene expression. This explains the more recent loss of sequence from MF1 (Tang et al., 2012). However, we are of course aware that there is many other contributing to variation in DNA methylation, including interdependent relationships between genic methylation and transcription (Zilberman et al., 2007). Additional data from a wider range of representative sub-taxa would help confirm these hypotheses based upon our initial survey.

In contrast to the complex pattern of methylation in different subgenomes, we found that methylation level in single-copy genes was universally higher than duplicated genes, with a correspondingly lower level of expression. These results are consistent with a lower level of transcription contributing to genes being more readily deleted due to lower fitness, so that they escape purifying selection (Diez et al., 2014). This further substantiates the hypothesis that epigenetic processes are a viable alternative mechanism for duplicated gene loss. Meanwhile, we find that the methylation level of single copy genes alone is good indicators of the methylation status of any given subgenome. Consistent with this, the methylation of the three ancestral sub-genomes of *B. oleracea* did not appear to be reflected at the level of the retained triplicate genes (Liu et al., 2014). The methylation and gene expression of retained single copy genes thus provide more credible residue markers of the intrinsic status of ancestral genomes, with biased gene loss during the formation and evolution of polyploids.

## Conclusion

We generated a representative whole genome methylation profile for the first time in *B. rapa* by using a modified RRBS method. We found that methylation level generally reflected the dominance of gene loss and gene expression between different ancestral subgenomes. The results here provide more evidence for the involvement of epigenetic mechanisms in polyploid genome evolution, as well as alternative mechanism for determining the fate of duplicated genes.

## Author contributions

The study was conceived by XC, XG, and KL. CT prepared the plant materials. XC and JW performed the experiments. XC and XG contributed to data analysis, bioinformatics analysis, and manuscript preparation. GK participated in writing the manuscript. All authors contributed to revising the manuscript. All authors had read and approved the final manuscript.

### Conflict of interest statement

The reviewer Maoteng Li declares that, despite having previously collaborated with the co-author Xianhong Ge, the review process was conducted objectively. The authors declare that the research was conducted in the absence of any commercial or financial relationships that could be construed as a potential conflict of interest.

## Supplementary material

Table S1 **Characterization of gene expression with different copies or within different subgenomes**.

Table S2 **Summary of methylated loci and annotation**.

Table S3 **The relative proportion of genes transcribed at high, medium and low levels in different tissues of ***B. rapa*****.

Figure S1 **The construction of a modified reduced representation bisulfite sequencing library**.

Figure S2 **The percentage of the three methylation contexts between whole genome and loci enriched using (***in silico***) mRRBS in (A) ***B. rapa*** and (B) rice (***Oryza sativa***)**.

Figure S3 **The ratio of genome-wide methylation levels for CG, CHG and CHH contexts**.

Figure S4 **Mean CHG methylation levels in different components of genic regions (A) within the three subgenomes and (B) between genes of different copy number**.

Figure S5 **Mean CHH methylation levels in different components of genic regions (A) within the three subgenomes and (B) between genes of different copy number**.

Figure S6 **Mean methylation level of two-copy genes in three subgenomes of ***B. rapa*****.

Figure S7 **Mean methylation level of three-copy genes in three subgenomes of ***B. rapa*****.

### Availability of supporting data

All the sequencing data used in this research have been submitted to public database NCBI under PRJNA281682.

## Abbreviations

RRBS: reduced representation bisulfite sequencing
NAA: a-naphthalenacetic acid
6-BA: 6-benzyl aminopurine
PE: paired-end
TSS: transcriptional start site
TRR: transcription termination region
RPKM: reads per kb per million reads
FPKM: fragments per kb per million reads
WGBS: whole-genome bisulfite sequencing
MSAP: methylation sensitive amplification polymorphism.
