# Supplementary material for: Genome-wide DNA methylation profiling by modified reduced representation bisulfite sequencing in Brassica rapa suggests that epigenetic modifications play a key role in polyploid genome evolution
Source: Front Plant Sci. 2015 Oct 9;6:836. doi: 10.3389/fpls.2015.00836 (PMC4598586; doi:10.3389/fpls.2015.00836)
Supplement: Table S3 — The relative proportion of genes transcribed at high, medium and low levels in different tissues of B. rapa. [file DataSheet3.DOCX]

Table S3. The relative proportion of genes transcribed at high, medium and low levels in different tissues of *B. rapa*

|  | Highly | | | | | | |  | Medium | | | | | | |  | Lowly | | | | | | |
| --- | --- | --- | --- | --- | --- | --- | --- | --- | --- | --- | --- | --- | --- | --- | --- | --- | --- | --- | --- | --- | --- | --- | --- |
|  | Gene copy | | |  | Sub-genome | | |  | Gene copy | | |  | Sub-genome | | |  | Gene copy | | |  | Sub-genome | | |
|  | Single | Two | Three |  | LF | MF1 | MF2 |  | Single | Two | Three |  | LF | MF1 | MF2 |  | Single | Two | Three |  | LF | MF1 | MF2 |
| Leaf^1^ | 7.3% | 10.4% | 14.4% |  | 10.2% | 9.2% | 9.8% |  | 30.7% | 37.0% | 34.0% |  | 35.5% | 31.6% | 32.7% |  | 62.0% | 52.6% | 51.6% |  | 54.3% | 59.2% | 57.5% |
| Silique^1^ | 8.8% | 12.5% | 17.4% |  | 12.4% | 10.9% | 11.5% |  | 32.1% | 38.6% | 33.6% |  | 36.6% | 32.8% | 33.9% |  | 59.1% | 48.9% | 49.0% |  | 51.0% | 56.3% | 54.6% |
| Leaf1^2^ | 5.2% | 8.2% | 13.5% |  | 8.0% | 7.5% | 7.7% |  | 25.9% | 36.1% | 32.4% |  | 32.8% | 28.5% | 29.9% |  | 68.8% | 55.7% | 54.2% |  | 59.2% | 64.0% | 62.4% |
| Leaf2^2^ | 6.3% | 10.0% | 15.1% |  | 9.5% | 8.7% | 9.3% |  | 25.9% | 35.6% | 31.8% |  | 32.5% | 28.5% | 29.3% |  | 67.7% | 54.4% | 53.0% |  | 58.0% | 62.9% | 61.4% |
| Root1^2^ | 5.4% | 9.5% | 16.3% |  | 9.0% | 8.5% | 8.9% |  | 29.0% | 41.6% | 35.8% |  | 37.0% | 32.7% | 33.1% |  | 65.6% | 49.0% | 48.0% |  | 54.0% | 58.8% | 58.0% |
| Root2^2^ | 6.6% | 10.9% | 17.1% |  | 10.1% | 9.7% | 10.1% |  | 28.9% | 41.3% | 36.2% |  | 37.0% | 32.5% | 33.1% |  | 64.5% | 47.9% | 46.7% |  | 52.9% | 57.8% | 56.9% |
| Stem^2^ | 6.3% | 10.3% | 16.5% |  | 9.8% | 9.1% | 9.7% |  | 29.7% | 40.1% | 34.1% |  | 36.7% | 31.9% | 32.9% |  | 64.0% | 49.6% | 49.3% |  | 53.5% | 59.1% | 57.4% |
| Flower^2^ | 5.5% | 8.7% | 14.1% |  | 8.5% | 7.8% | 8.1% |  | 30.4% | 41.8% | 38.2% |  | 38.3% | 33.5% | 34.4% |  | 64.1% | 49.5% | 47.7% |  | 53.2% | 58.7% | 57.5% |
| Silique^2^ | 6.0% | 8.5% | 14.2% |  | 8.6% | 7.9% | 8.4% |  | 29.3% | 38.7% | 33.3% |  | 35.7% | 31.1% | 32.1% |  | 64.8% | 52.8% | 52.6% |  | 55.7% | 61.0% | 59.5% |
| Callus^2^ | 5.3% | 8.4% | 13.7% |  | 8.3% | 7.3% | 7.9% |  | 26.0% | 36.9% | 34.7% |  | 33.0% | 30.2% | 30.4% |  | 68.7% | 54.7% | 51.6% |  | 58.7% | 62.5% | 61.8% |
| Average^1^ | 8.1% | 11.5% | 15.9% |  | 11.3% | 10.0% | 10.7% |  | 31.4% | 37.8% | 33.8% |  | 36.1% | 32.2% | 33.3% |  | 60.6% | 50.7% | 50.3% |  | 52.6% | 57.8% | 56.0% |
| Average^2^ | 5.8% | 9.3% | 15.0% |  | 9.0% | 8.3% | 8.8% |  | 28.1% | 39.0% | 34.6% |  | 35.4% | 31.1% | 31.9% |  | 66.0% | 51.7% | 50.4% |  | 55.7% | 60.6% | 59.4% |

^1^ indicates tissues from 3H120 accession, with expressed genes were grouped into low (RPKM<=5), medium (5<RPKM<=50) and highly (RPKM>50) transcribedgenes. ^2^ indicates tissues from accession Chiifu-401-42 and expressed genes were grouped into low (FPKM<=5), medium (5<FPKM<=50) and highly (FPKM>50) transcribedgenes (Tong et al., 2013).
