# Supplementary material for: Genome-wide DNA methylation profiling by modified reduced representation bisulfite sequencing in Brassica rapa suggests that epigenetic modifications play a key role in polyploid genome evolution
Source: Front Plant Sci. 2015 Oct 9;6:836. doi: 10.3389/fpls.2015.00836 (PMC4598586; doi:10.3389/fpls.2015.00836)
Supplement: Figure S1 — The construction of a modified reduced representation bisulfite sequencing library. [file Image1.PDF]

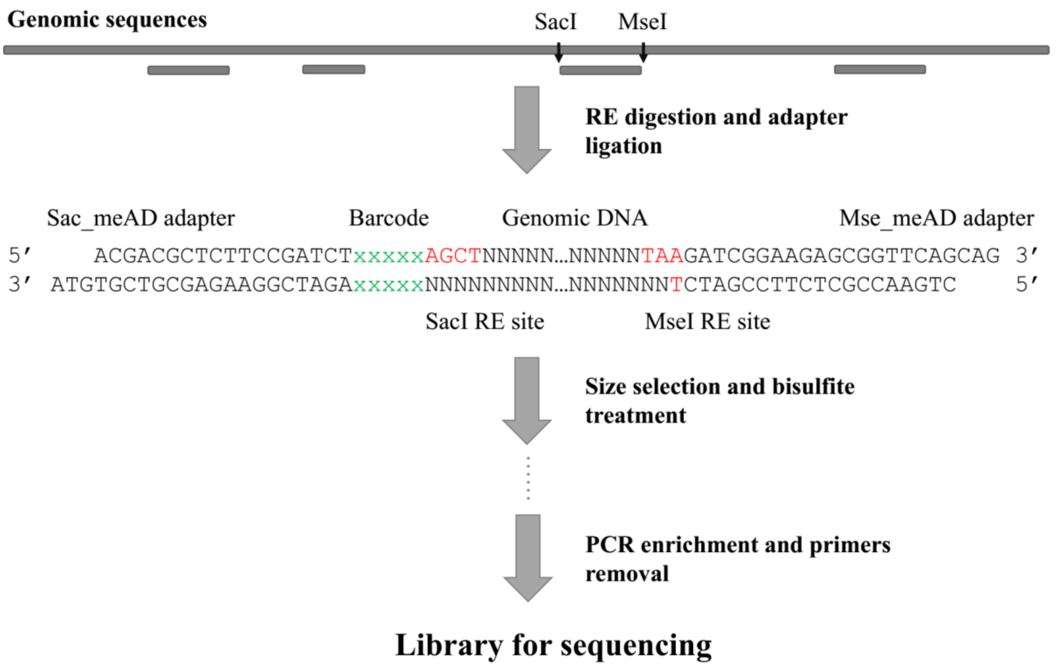

Figure S1. The construction of a modified reduced representation bisulfite sequencing library. Adapter sequences were truncated and all cytosines were methylated, and forwardac_meAD and Mse_meAD adapter sequences were 5’-phosphorylated. Illumina PCR primers used were from previous work (Chen et al., 2013).

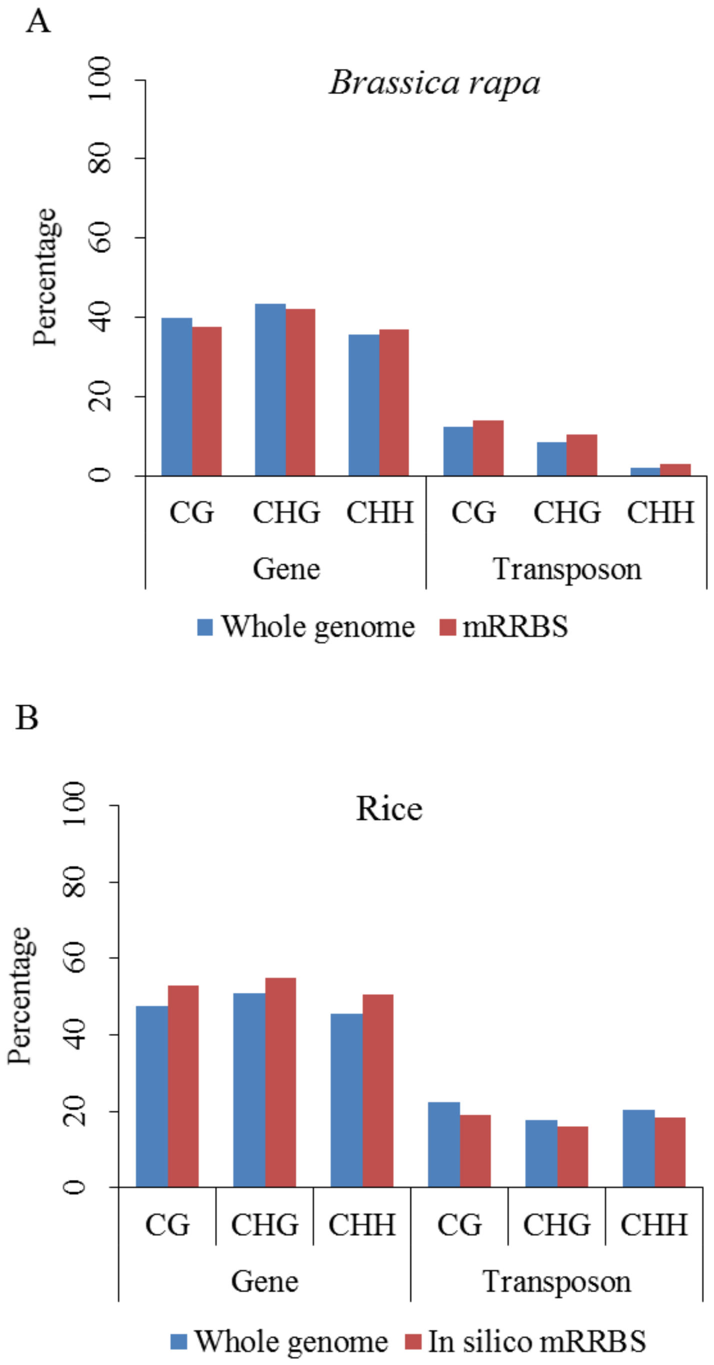

Figure S2. The percentage of the three methylation contexts between whole genome and loci enriched using (*in silico*) mRRBS in (A) *B. rapa* and (B) rice (*Oryza sativa*).

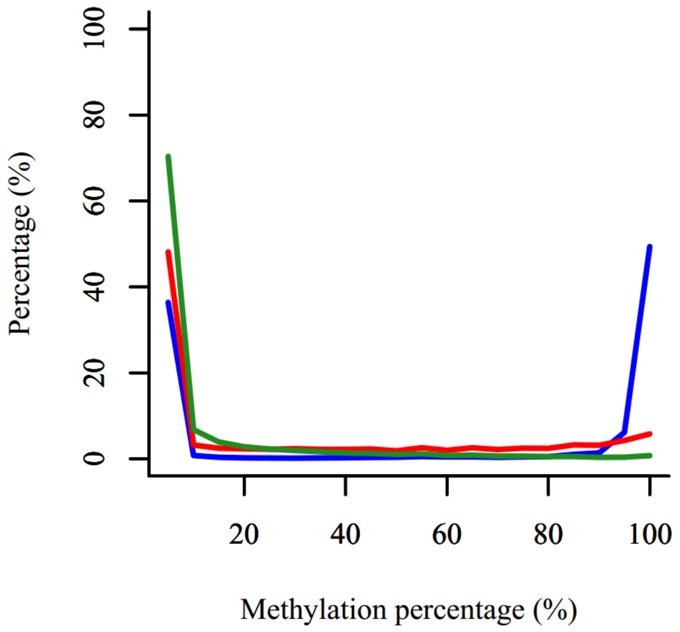

Figure S3 The ratio of genome-wide methylation levels for CG, CHG and CHH contexts. Blue: CG; Red:CHG; Green:CHH.

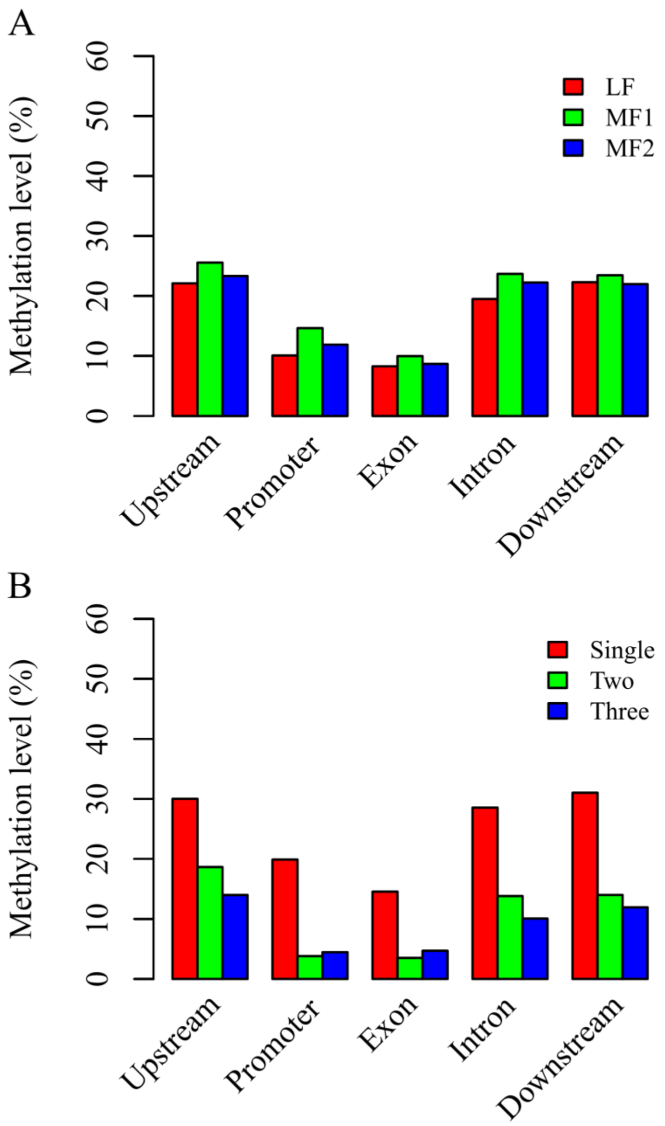

Figure S4. Mean CHG methylation levels in different components of genic regions (A) within the three subgenomes and (B) between genes of different copy number.

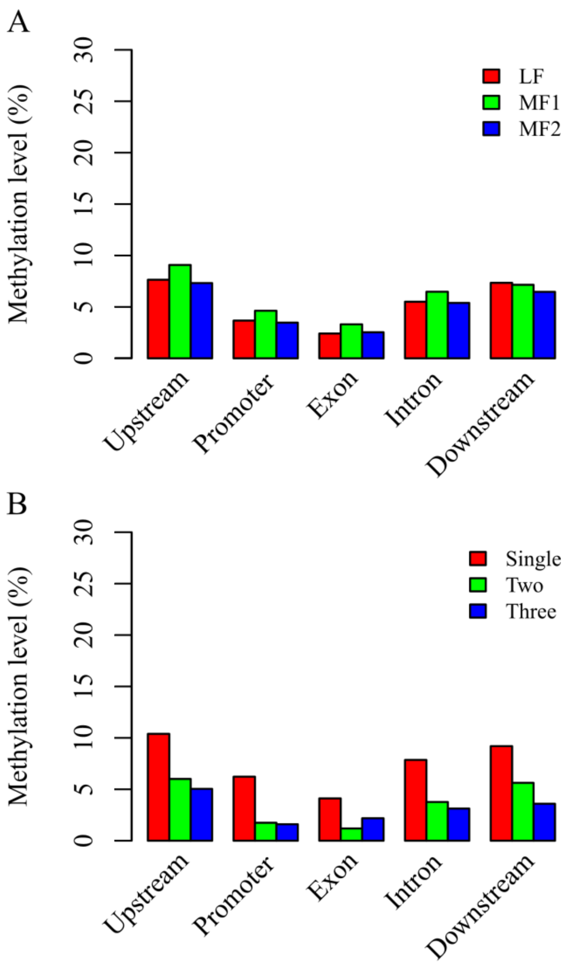

Figure S5. Mean CHH methylation levels in different components of genic regions (A) within the three subgenomes and (B) between genes of different copy number.

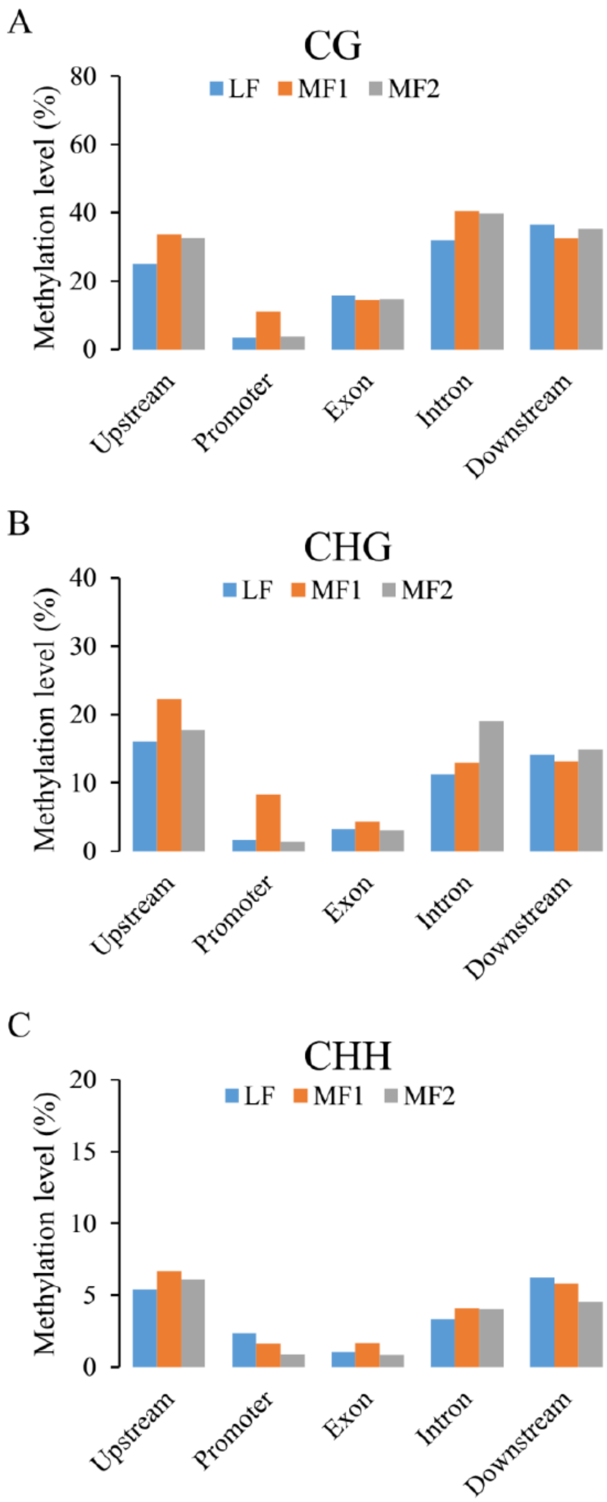

Figure S6. Mean methylation level of two-copy genes in three subgenomes of *B. rapa*.

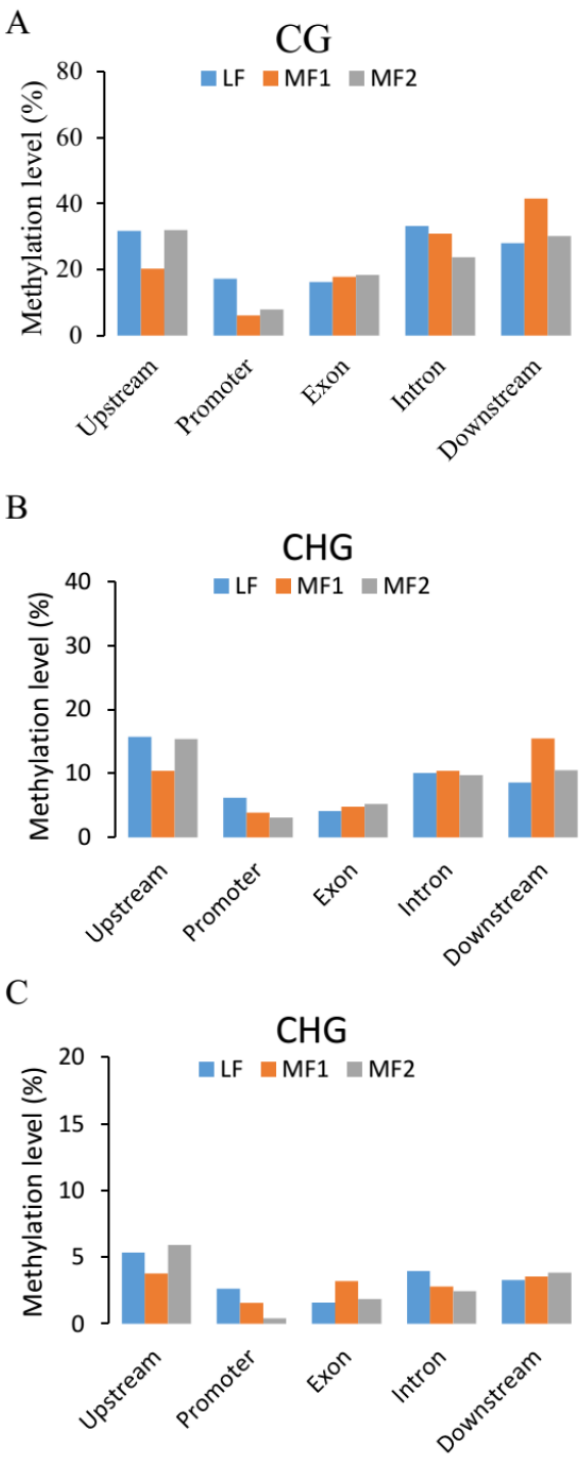

Figure S7. Mean methylation level of three-copy genes in three subgenomes of *B. rapa*.
